# NLRP3 inflammasome activation contributes to the cognitive decline after cardiac surgery

**DOI:** 10.3389/fsurg.2022.992769

**Published:** 2022-11-02

**Authors:** Gang Ma, Ping Sun, Yi Chen, Xin Jiang, Caixia Zhang, Baofu Qu, Xiangkun Meng

**Affiliations:** ^1^ Department of Anaesthesiology, General Hospital of Ningxia Medical University, Yinchuan, China; ^2^ Department of Anaesthesiology, Ningxia Medical University, Yinchuan, China; ^3^ Department of Gastroenterology, General Hospital of Ningxia Medical University, Yinchuan, China

**Keywords:** NLRP3 inflammasome, perioperative neurocognitive disorders (PND), inflammation, interleukin - 1β, interleukin

## Abstract

**Background:**

Perioperative neurocognitive disorders (PND) are a common complication of cardiac surgery in elderly patients. The etiopathogenesis of PND is not clear. Nod-like receptor family pyrin domain containing 3 (NLRP3) inflammasome, a macromolecular protein complex, regulates inflammation by inducing the release of proinflammatory cytokines interleukin (IL)-1β and IL-18. Studies have demonstrated a close link between the NLRP3 inflammasome and central nervous system diseases. Nevertheless, the involvement of NLRP3 inflammasome in the causation of PND occurring after cardiac surgery is unclear. This study aimed to investigate the association of serum NLRP3 level with PND.

**Methods:**

We performed a retrospective study, enrolled 75 patients undergoing elective cardiac surgery and evaluated their cognitive functions one day before and 7 days after surgery. PND were determined according to the International Study of Postoperative Cognitive Dysfunction studies. Demographics and perioperative parameters were recorded. Perioperative serum NLRP3 protein, IL-1β, and IL-18 levels were monitored.

**Results:**

The PND incidence in our cohort was 33.33%. NLRP3 protein levels were significantly increased in all patients at each postoperative time-point after general anesthesia and cardiac surgery under cardiopulmonary bypass. Patients showing cognitive dysfunction had higher serum NLRP3 protein, caspase-1, IL-1β, and IL-18 levels immediately after the operation. Variables associated with the incidence of early PND were included in the regression models. After adjusting for confounding variables, high serum NLRP3 protein level at the end of the operation and old age were identified as independent predictors of PND.

**Conclusions:**

High serum NLRP3 protein level at the completion of cardiac surgery was associated with a higher risk of PND seven days after surgery.

**Trial registration:**

The study was registered at Clinicaltrials.gov (registration number: NCT04191642).

## Background

Cardiopulmonary bypass (CPB) during cardiovascular surgery facilitates the operation and helps sustain the patient's life. However, systemic inflammatory response induced by CPB contributes to a number of complications (1), including perioperative neurocognitive disorders (PND). PND are characterized by short-term or long-term decline in cognitive performance after surgery affecting different aspects of cognition (e.g., impaired visual or verbal memory, attention, language understanding, concentration), and thus increasing mortality and impairing the quality of life (2). Because of the major physiological impact of cardiac surgery, the incidence of PND is particularly high after cardiac surgery, with a reported incidence of 25%–50% within one week after coronary artery bypass grafting (3, 4). Although the pathogenetic mechanisms of PND are not well characterized, inflammation is believed to play a potential role in its causation. Surgical trauma provokes central nervous system and systemic inflammation, triggering the release of inflammatory mediators such as interleukin (IL)-1β. Neuroinflammation induced by surgical trauma can occur due to passage of inflammatory mediators across the blood–brain barrier or due to trauma-induced secretion of inflammatory cytokines within the brain, leading to cognitive decline. However, the pathogenesis of PND is a complex phenomenon that likely involves the interaction between multiple factors. Therefore, investigation of the specific role of inflammation in the pathogenesis of PND is a key imperative.

The inflammasome, a key element of the innate immune system, is a macromolecular protein complex that modulates inflammation. The Nod-like receptor family pyrin domain containing 3 (NLRP3) inflammasome, as the most-characterized inflammasome till date, is a platform where cysteine protease caspase-1 is activated. Caspase-1 initiates the production of proinflammatory cytokines IL-1β and IL-18. Increased level of NLRP3 has a high positive predictive value for diagnosing various disorders and is a determinant of short- and long-term prognoses (5, 6). Recent research has indicated a strong link between the NLRP3 inflammasome and central nervous system diseases (6, 7). Targeting the assembly and function of the NLRP3 inflammasome is a novel therapeutic strategy for inflammatory diseases including ischemic stroke concomitant with diabetes (7). The NLRP3 inflammasome has been identified as a potential therapeutic target and biomarker in the management of traumatic brain injury (6).

An animal experiment suggested the involvement of NLRP3 priming status in the brains of old mice in the causation of isoflurane-induced cognitive impairment and hippocampal inflammation (8). To the best of our knowledge, the involvement of the NLRP3 inflammasome in operation-induced PND is not well characterized. In our animal experiment, the upregulation of systemic and hippocampal NLRP3 expression after surgery in aged mice was found to be associated with memory and learning dysfunction (data not published). However, the relationship between cognitive dysfunction after surgical trauma and the NLRP3 expression in humans remains to be investigated.

Considering the above body of evidence, we designed this clinical study to test our hypotheses that the NLRP3 inflammasome participates in the incidence of PND and that the occurrence of PND after cardiac surgery is attributable to an increase in perioperative NLRP3 level. This study may provide evidence to develop specific drugs or therapies to reduce NLRP3-induced cognitive impairment-associated PND and to improve prognosis.

## Materials and methods

### Subjects

This study was approved by the Ethics Committee of the General Hospital of Ningxia Medical University (No. 2018-020). Written informed consent was obtained from all patients. The study was conducted in compliance with the principles of the *Declaration of Helsinki*. Patients receiving heart valve replacement at the General Hospital of Ningxia Medical University between May 2019 and March 2021were eligible for enrolment. The inclusion criteria were: patients aged ≥18 years who were scheduled for aortic or mitral valve replacement under moderate hypothermic CPB; minimum expected postoperative length of hospital: 7 days; American Society of Anesthesiologists physical status II or III. The exclusion criteria were: (1) incomplete cognitive assessment or a Mini-mental state examination (MMSE) score <18; (2) complications that may affect cognitive assessment, such as unstable mental status, mental illness, or language, visual or auditory dysfunction; (3) left ventricular ejection fraction (LVEF) <45%; (4) patients who received high-dose pharmacologic intervention (phenylephrine 100-µg bolus, and epinephrine >0.1 mg/kg/min) for hemodynamic stability [mean arterial pressure (MAP) >60 mmHg]. A nonsurgical control group (spouses who provided written informed consent and qualified the same inclusion/exclusion criteria) was established to determine the effects of repeated neuropsychological testing (practice effect). This study was registered at Clinicaltrials.gov (NCT04191642).

### Anesthesia and surgery

Standard general anesthesia was applied throughout. Nasopharyngeal and rectal temperatures, 5-lead electrocardiogram, capnography, and pulse oximetry were routinely monitored. Systemic arterial blood pressure was detected by radial artery catheterization. Anesthesia was initiated using target-controlled infusion of propofol, and the bispectral index was maintained at 40–60. In the unconscious state, 0.2 mg/kg cisatracurium and 0.8–1.5 µg/kg sufentanil were infused. After tracheal intubation, the lungs were ventilated with O_2_-rich air (0.6 of inspired O_2_) calibrated to an end-tidal CO_2_ partial pressure of 35–45 mmHg. Then, the central venous pressure and fluid control were monitored by inserting a central venous catheter. Intermittent IV boluses of sufentanil were administered (total dose, 3–5 µg/kg) according to the blood pressure and heart rate. All patients received infusion of 0.1 mg/kg/h cisatracurium throughout the surgery.

Standard median sternotomy was performed for surgical approach in all patients. The body temperature was reduced to 30–32°C during CPB. All patients were treated with an intermittent antegrade infusion of cooled high-potassium blood to induce cardioplegia during continuous aortic cross-clamping (ACC) using a non-pulsatile flow rate of 2.2–2.8 L/min/m^2^. In a cell-saving device, the blood in the CPB circuit and from the surgical field was obtained, centrifuged, washed, and infused within 4 h after CPB. Hematocrit was maintained at >25%, with the addition of blood, if required. To normalize serum blood glucose level >150 mg/dl, insulin therapy was started perioperatively. Pre- or post-CPB hemodynamic modulation was targeted to maintain MAP at 60–100 mmHg. Hypertension was handled with sufentanil (bolus dose) or 0.1–0.5 mg/kg/min nitroglycerin, or both. Hypotension was treated with fluid intake (including crystalloids, colloids, and blood products) or use of vasoactive drugs (phenylephrine IV, 20–100 µg, or concomitant epinephrine, 0.01–0.1 mg/kg/min). The dosage of vasoactive drugs, fluid intake, and urine were recorded after the surgery.

Prior to skin closure, 0.08 mg/kg midazolam (bolus dose) was administered intravenously and the infusion of propofol was terminated. Postoperatively, all patients were admitted to the intensive care unit (ICU). Extubation was performed when a patient was able to maintain adequate spontaneous breathing and required the least oxygen support, which was indicated by normal arterial blood gas levels. When a patient did not require inotropic or oxygen and was hemodynamically stabilize with normal blood gas variables, he/she was discharged from the ICU. The length of the ICU stay was recorded.

Basic data collection included demographic variables obtained *via* questionnaire, including sex, age, years of education, and body mass index (BMI). Medical history data acquired from patient medical records included history of hypertension, hyperlipidemia, diabetes, aortic plaque, chronic renal dysfunction, cerebrovascular disease, and carotid artery stenosis. Hemodynamic measurements were performed before anesthesia initiation, before skin incision, after sternotomy, CBP-cessation, and at 1, 2 and 4 h after CPB (post-CPB 1, 2, 4 h respectively). At pre-incision, CBP-cessation, and post-CPB 2 h and 4 h, radial artery blood sample was collected for blood gas, lactic acid, and blood glucose examinations. Peripheral venous blood sample was collected to determine IL-1β, IL-18, and NLRP3 expressions at pre-induction, at the end of the operation, and post-CPB 3 d. Complications occurring within seven days postoperatively (i.e., infection, bleeding, and organ dysfunction) were recorded.

### Neuropsychological assessment

Cognitive function assessments were performed 1 day before and 7 days after the operation in a quiet environment in the general ward. A widely-used test battery (9) was performed in Chinese. The specific tests conducted were: MMSE; Trail Making Test (A/B); WAIS Digit Symbol Substitution Test (WDSST); Rey Auditory Verbal Learning Test (RAVLT; immediate and delayed recall); brief visuospatial memory test-revised (BVMT-R); Controlled Oral Word Association Test (COWAT); and WAIS Digit Span Test (forward and backward). At 1 week post-operatively, the cognitive tests were repeated. Pain was assessed using the Visual Analog Scale (VAS) at 7 days postoperatively.

PND were defined using *z* scores as recommended by the International Study of Postoperative Cognitive Dysfunction studies (10). To examine the learning effect, we calculated the variations (including mean and standard deviation) from baseline in each test performance for controls. For the patients, we compared the scores before and at 1-week after the operation, subtracted the average learning impact, and divided the results by the standard deviation (SD) of the control group to determine the *z* score for each test. Then, a composite *z* score was computed as the sum of these *z* scores for any patient divided by the standard deviation of the corresponding sum in the controls. Then, to create a combined *z* score, the *z* scores of all tests in a patient were added and divided by the SD for this sum of *z* scores in the controls. PND was diagnosed if the *z* score was ≥1.96 in ≥2 separate cognitive tests or the composite *z* score was ≥1.96. Postoperative delirium was also considered as PND.

### Serum NLRP3,IL-1β, and IL-18 levels

After 10 min of centrifugation at 2,000 *g*, serum was obtained and stored at −80°C until further processing. Enzyme-linked immunosorbent assay (ELISA) kits (CLOUD-CLONE CORP., USA) were used to detect plasma levels of IL-18 (L200910330580), IL-1β (L200910324), and NLRP3 (L2009103305830), according to the manufacturer's protocols.

### Endpoints and sample size

Our primary objective was to compare NLRP3 levels between patients with and without PND. The secondary objective was to compare serum IL-18 and IL-1β levels. In line with a previous study (11) and our pilot study, we assumed that 40% of the patients are affected by PND and that the postoperative NLRP3 levels of PND patients will be three times higher than those of non-PND patients. Factoring a two-tailed significance level of 0.05, a sample size of 71 was required to obtain a 90% statistical power to detect this difference. Given an estimated attrition rate of 10%, the final sample size was increased to a total of 81 patients.

### Statistical analysis

Categorical variables were reported as frequency (percentage) and continuous variables as mean ± SD. Normality of distribution of continuous variables was assessed using the Kolmogorov–Smirnov test. Between-group differences with respect to normally-distributed variables were assessed using Student's *t*-test, while those with respect to non-normally distributed variables were assessed using the non-parametric Mann-Whitney test. Categorical variables were analyzed using Chi-squared test and Fisher's exact test. Differences in NLRP3, IL-1β, IL-18 levels, and other vital parameters over time between the PND and the non-PND groups were assessed using repeated measures analysis of variance (ANOVA) with *post hoc* Bonferroni correction.

Association of serum NLRP3 level with the onset of early PND was assessed using multivariate logistic regression analysis and the results presented as odds ratio (OR) and 95% confidence interval (CI). First, association of each baseline or perioperative variable with PND was tested. Variables associated with *P* values ≤0.05 were included in multivariate logistic regression to identify the risk-adjusted predictors of PND. Relationships of NLRP3 with IL-1β and IL-18 was assessed using Pearson correlation analysis. Two-tailed *P* values <0.05 were considered indicative of statistical significance. All statistical analyses were conducted using SPSS 22.0 (SPSS Inc, Chicago, Illinois).

## Results

**Figure 1:**
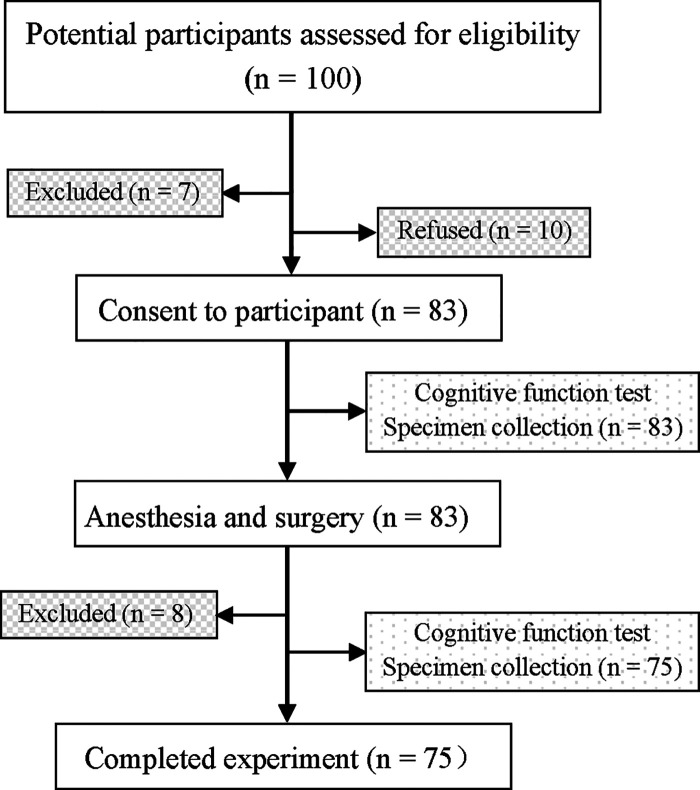
Flow chart of the study. overall, 100 patients were recruited, and 75 patients completed all the tests.

Ninety-three of the initial 100 patients qualified the selection criteria, of whom 10 opted out of the study. The remaining 83 patients provided informed consent and were enrolled. Of them, 8 patients did not complete all follow-up tests because of: withdrawal of consent due to no postoperative NLRP3 value or no postoperative cognitive testing (*n* = 4), or hemodynamic instability (*n* = 3), and one death caused by intractable ventricular fibrillation on the second day after surgery. Finally, 75 patients were included (Figure 1), and their characteristics are summarized in Table 1. To estimate the size of the practice effect for neuropsychological tests, we enrolled 21 control subjects during the same period, who were matched with the patients with respect to many parameters, such as age, proportion of females, education level, and BMI. Descriptive details of the changes in each cognitive test are shown in Table 2.

**Table 1:** Characteristics of the study population.

Variables	Non-PND (*n* = 50)	PND (*n* = 25)	*P*-value
Male/Female	28/22	12/13	0.341
Age (years)	57.33 ± 7.65	62.65 ± 6.94	0.004
BMI (kg/m^2^)	23.35 ± 2.60	23.83 ± 3.08	0.480
Education (years)	7.43 ± 3.39	4.54 ± 3.93	0.001
Hypertension	17/33	13/12	0.134
Diabetes	3/47	3/22	0.652
Preoperative LVEF (%)	61.08 ± 6.56	60.58 ± 7.11	0.76
CPB time (min)	131.67 ± 40.61	151.81 ± 48.08	0.059
ACC time (min)	89.90 ± 32.28	111.42 ± 44.96	0.019
Surgery time (min)	243.35 ± 51.75	268.96 ± 55.11	0.050
Extubation time after surgery (h)	11 ± 2	10 ± 2	0.78
Postoperative ICU stay (days)	2.47 ± 1.12	3.85 ± 1.87	0.000
VAS score	2.35 ± 1.28	2.31 ± 1.12	0.896
Occurrence of postoperative complications (within 7 days)	2 (pulmonary infection)	1 (wound infection)	0.998

Data presented as mean ± standard deviation or ratio (the same in other tables).

PND, perioperative neurocognitive disorders; BMI, body mass index; LVEF, left ventricular ejection fraction; CBP, cardiopulmonary bypass; ACC, aortic cross-clamping; VAS, visual analog scale.

**Table 2:** Cognitive performance of patients and control subjects.

Cognitive tests	Control subjects (*n* = 21)		Non-PND (*n* = 50)		PND (*n* = 25)	
	Baseline	After 7 days	Baseline	Postoperative	Baseline	Postoperative
MMSE (score)	23.14 ± 2.73	23.38 ± 2.73	24.18 ± 2.58	23.51 ± 2.72	22.96 ± 2.25	19.85 ± 2.17
RAVLT (immediate)	14.86 ± 4.36	15.19 ± 4.20	14.43 ± 4.51	15.08 ± 4.07	14.69 ± 3.52	11.92 ± 3.33
RAVLT (delayed)	27.86 ± 2.71	28.90 ± 2.56	28.55 ± 2.44	28.41 ± 2.49	27.19 ± 2.10	23.46 ± 2.02
BVMT-R	9.86 ± 1.35	10.48 ± 1.40	9.57 ± 1.53	10.20 ± 1.50	10.96 ± 1.37	8.8 ± 1.03
Trail Making A	74.38 ± 23.67	68.00 ± 22.93	69.00 ± 21.98	71.14 ± 22.25	91.31 ± 36.13	115.04 ± 41.32
Trail Making B	93.43 ± 19.31	85.05 ± 18.04	91.98 ± 26.02	90.43 ± 25.55	116.08 ± 26.83	139.50 ± 27.76
WAIS Digit Span	8.00 ± 1.41	8.10 ± 1.04	8.57 ± 1.34	8.49 ± 1.16	7.54 ± 0.99	6.46 ± 0.86
WDSST	15.33 ± 2.69	16.29 ± 2.78	16.96 ± 3.03	16.02 ± 3.31	13.81 ± 1.92	10.46 ± 1.92
COWAT	39.24 ± 5.24	40.29 ± 4.82	41.20 ± 4.90	41.43 ± 4.83	35.58 ± 4.66	29.42 ± 3.92

Data are shown as mean ± standard deviation.

PND, perioperative neurocognitive disorders; MMSE, mini-mental state examination; WDSST, WAIS digit symbol substitution test; RAVLT, Rey auditory verbal learning test; BVMT-R, brief visuospatial memory test-revised; COWAT, controlled oral word association test.

### Patient characteristics

Two patients suffered delirium after the surgery, according to their clinical features and the CAM–ICU scores, their conditions were promptly resolved after drug treatment. According to the neuropsychological assessment at seventh day postoperatively (Table 2), cognitive impairment was identified in 25 patients (including the two patients with delirium), but in none of the control subjects. Therefore, the incidence of PND in our cohort was 33.33% (25/75). There were no significant differences between patients with and without PND with respect to physiological parameters or laboratory markers (e.g., MBP, HR, SPO_­2_, body temperature, lactic acid, blood glucose). Several indices, including the sex distribution, hypertension, BMI, occurrence of postoperative complications, and preoperative LVEF, were comparable in the PND and non-PND groups (*P *> 0.05; Table 1). Patients with PND were significantly older (57.33 ± 7.65 vs. 62.65 ± 6.94 years, *P* = 0.004) and less educated (7.43 ± 3.39 vs. 4.54 ± 3.93 years, *P* = 0.001) than the non-PND subjects. Regarding perioperative parameters, the PND group relative to the non-PND group had longer ACC time (111.42 ± 44.96 vs. 89.90 ± 32.28 h, *P *= 0.019) and postoperative ICU stay (3.85 ± 1.87 vs. 2.47 ± 1.12 days, *P* < 0.001).

### Serum NLRP3, caspase-1, IL-1β and IL-18 profiles

**Figure 2:**
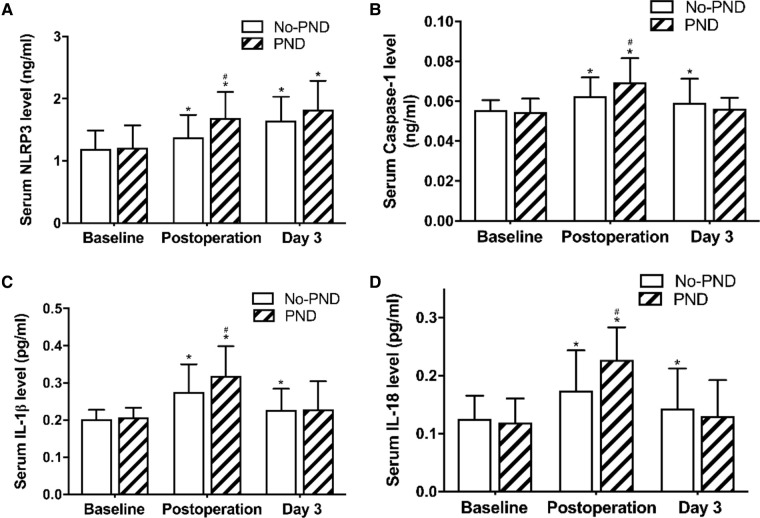
Serum NLRP3, caspase-3, IL-18, and IL-1β in non-PND and PND groups. * *P <* 0.05 vs. baseline; ^#^ *P <* 0.05 vs. the non-PND group at same time-point. A moderate positive association between NLRP3 and IL-1β (*r* = 0.330, *P* = 0.004) levels and a weak positive association of increase in NLRP3 with increase in IL-18 (*r* = 0.265, *P* = 0.021) and caspase-1 (*r* = 0.241, *P* = 0.038) levels was observed at the end of the surgery (Figure 3). Abbreviations: NLRP3, Nod-like receptor family pyrin domain containing 3; PND, Perioperative neurocognitive disorders; IL, interleukin.

We compared the NLRP3 inflammasome-related proteins between the two groups. Serum NLRP3 levels were low before surgery. General anesthesia and cardiac surgery under CPB significantly increased NLRP3 protein levels at each postoperative time-point in both PND group (1.69 ± 0.41, 1.82 ± 0.47 vs.1.21 ± 0.36 ng/ml, *P *< 0.001) and non-PND group (1.38 ± 0.36, 1.65 ± 0.39 vs. 1.19 ± 0.30 ng/ml, *P *< 0.001, Figure 2A).

**Figure 3:**
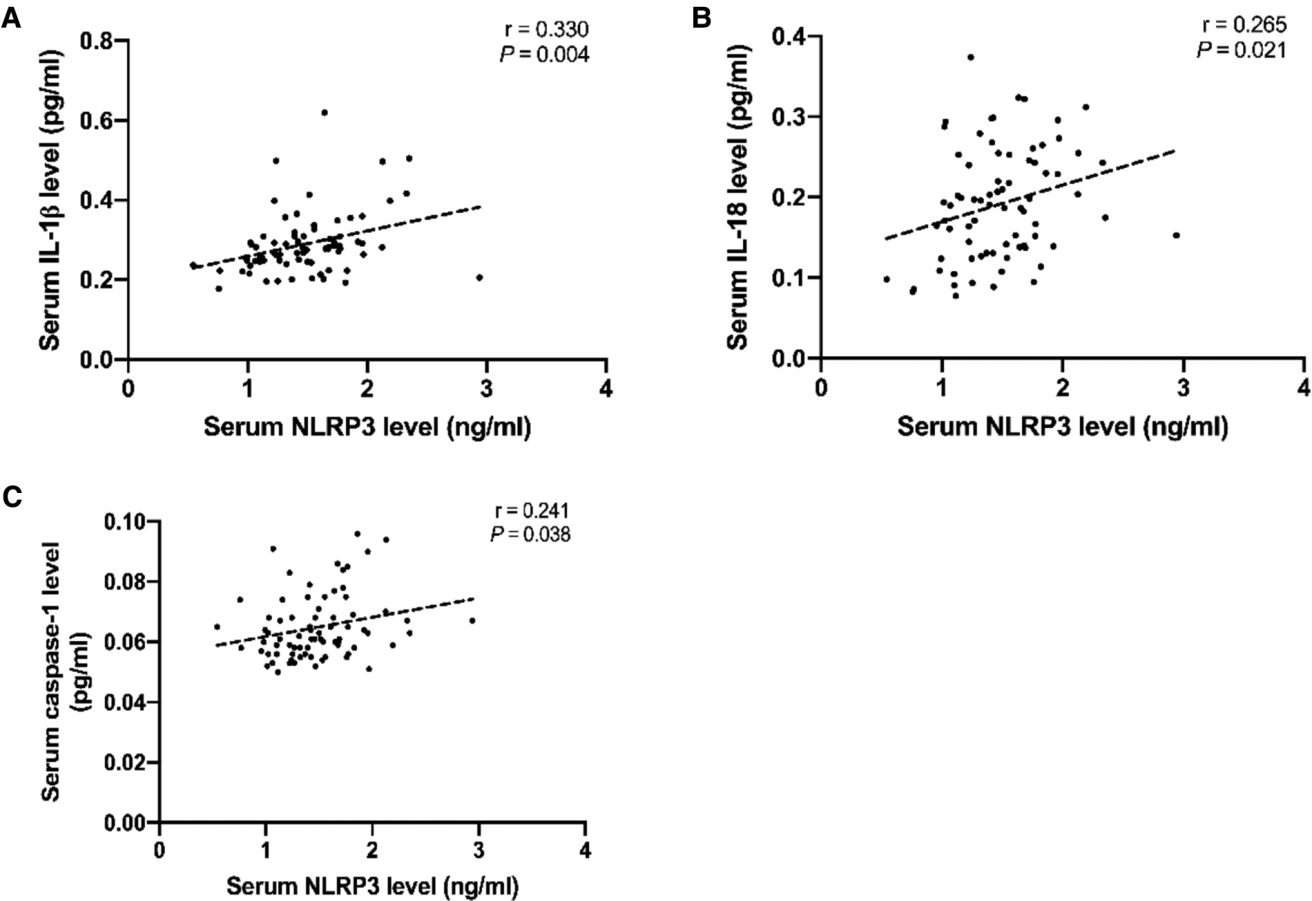
Scatter plots showing the positive relationship of NLRP3 with IL-1β (**A**), IL-18 (**B**), and caspase-1 (**C**). Dotted lines present the trend lines. Abbreviations: NLRP3, Nod-like receptor family pyrin domain containing 3; IL, interleukin.

There were no significant between-group differences with respect to preoperative serum NLRP3 levels (Figure 2A). However, immediately after the operation, the levels were significantly higher in early PND patients than in the non-PND patients (1.69 ± 0.41 vs. 1.38 ± 0.36 ng/ml, *P *= 0.001, Figure 2A).

Corresponding to the rising level of NLRP3, serum caspase-1, IL-1β, and IL-18 levels were elevated postoperatively and reached peak level immediately after the operation, and then decreased at day 3 postoperatively (Figures 2B–D), but remained higher than the baseline levels. Compared with the non-PND patients, PND patients had higher levels of caspase-1, IL-1β, and IL-18 immediately after the operation (*P *= 0.009, 0.021, and 0.001, respectively, Figures 2C,D).

## Variables associated with occurrence of early PND

Variables associated with the occurrence of early PND were included in the regression models. After adjusting for potential confounding factors, high serum NLRP3 protein level at the end of the operation was found to be an independent predictor of cognitive dysfunction (OR 0.128, 95% CI, 0.021–0.763, *P* = 0.024; Table 3). Old age was another independent predictor of early PND (OR 0.910, 95% CI, 0.021–0.763, *P* = 0.040; Table 3).

**Table 3:** Predictors of postoperative cognitive dysfunction at 1 week after surgery.

Risk factor	Univariate analyses^a^, *P*	Multivariate logistic regression analysis^b^	
		OR (95% CI)	*P*
Age	0.017	0.910 (0.021, 0.763)	0.042
Education	0.011	1.198 (0.997, 1.440)	0.054
ACC Time	0.033	0.982 (0.964, 1.001)	0.070
ICU stay	0.030	0.737 (0.47, 1.163)	0.184
Serum NLRP3 level (Immediately after the operation)	0.024	0.128 (0.021 0.763)	0.024
Serum IL-18 level (Immediately after the operation)	0.034	0.042 (0.000, 40.526)	0.533

NLRP3, Nod-like receptor family pyrin domain containing 3; ICU, intensive care unit; CI, confidence interval; OR, odds ratio; ACC, aortic cross-clamping; IL, interleukin.

^a,b^ Occurrence of postoperative cognitive dysfunction was modeled as a function of a single predictor and as a function of all significant predictors in the univariate analyses (*P* ≤ 0.05) respectively.

## Receiver operating characteristic (ROC) analysis

**Figure 4:**
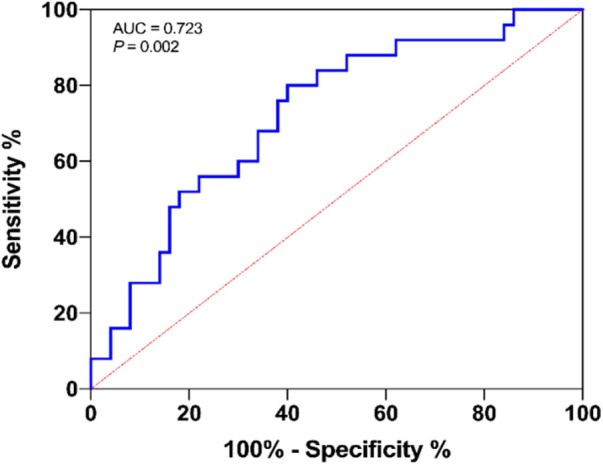
ROC curve showing the predictive ability of NLRP3 for PND in cardiac operation patients.

On ROC curve analysis, NLRP3 protein levels at the end of the operation showed a good predictive accuracy for PND (area under the curve = 0.723; 95% CI, 0.603–0.843; *P *= 0.002; Figure 4).

## Discussion

There were two main findings of our study. (1) Serum NLRP3 protein, IL-18, and IL-1β levels increased after cardiac surgery; and (2) elevated NLRP3 and old age were associated with higher risk of PND after cardiac surgery. These results seem to confirm our hypothesis that the NLRP3 inflammasome plays an instrumental role in the development of neurocognitive deficit after surgery and may be a predictor of PND following cardiac surgery.

The neuropsychological test battery and the definition of PND applied in this study have been widely used in previous studies (9, 12), including the assessment of attention, concentration, executive function, memory, psychomotor speed, and visuospatial ability. Though these disorders may also occur after noncardiac surgeries, they are a special concern after cardiac surgery because of perturbations, such as CPB, median sternotomy, and long surgical and anesthetic time (13). Elimination of some factors (e.g., poor cardiac function, history of mental illness) that may have influenced the results helped improve the credibility of our results. In our study, the incidence of new PND within 7 days after surgery was 33.33%. These statistics, despite being higher than the incidence rate after noncardiac surgery, are consistent with other research in cardiac surgery (14).

Surgical trauma can initiate a systemic inflammatory response, and inflammatory cytokines can cause neuroinflammation by triggering the release of inflammatory cytokines in the brain or by directly crossing the blood–brain barrier, contributing to cognitive deterioration. To verify the pro-inflammatory characteristics of NLRP3, we focused on the role of the NLRP3 inflammasome in PND occurrence. As evidenced by the increased levels of IL-1β and IL-18 and significant upregulation of the NLRP3 protein, our findings clearly demonstrate that anesthesia and/or cardiac surgery can induce severe inflammatory response.

Based on the behavioral performance after surgery, we divided the patients into PND and non-PND groups. PND were clearly identified as a multi-factor condition. Our results indicate higher risk factors in the PND group, including ACC time, poor education, postoperative ICU stay, surgery time, and serum NLRP3 and IL-1β levels at the end of the operation. The increased NLRP3, IL-1β, and IL-18 levels in the PND patients are consistent with previous reports which showed that inflammation, particularly increased IL-1β level, is instrumental in PND occurrence (2, 15). More importantly, multiple logistic regression analyses and correlation analysis identified high serum NLRP3 level at the end of surgery was associated with a higher risk of cognitive dysfunction seven days after heart surgery. This indicates the involvement of NLRP3 inflammasome in the pathogenesis of PND in a clinically complex environment. These findings are consistent with another study in which NLRP3 inflammasome activation was found to be linked with inflammation-induced cognitive dysfunction and neuropathological variations with aging (16). In another study, patients with acute coronary syndrome showed increased peripheral blood monocyte NLRP3 protein level, which showed a correlation with the severity of coronary atherosclerosis. This suggested its potential prognostic relevance in predicting major adverse cardiac events (5). These studies indicate that NLRP3 can be a predictor of inflammation-related diseases. Our ROC curve analysis also suggested that serum NLRP3 level is a good predictor of PND. In addition, since the ablation of each part of the NLRP3 inflammasome helps prevent age-related cognitive decline caused by neuroinflammation and neurodegeneration, such as in Alzheimer's disease (16–18), we deduce that the NLRP3 inflammasome can be immensely valuable as a potential therapeutic target for PND.

Surgery can activate immune cells, thus amplifying the immune response. In this process, the NLRP3 inflammasome modulates caspase-1 activation, which determines the maturation and production of IL-1β and IL-18. IL-1β is known to play an important role in the pathological process of PND, and IL-18 can influence the integrity of neurons and increase neuroinflammation in the brain (19), thus leading to cognitive deterioration in Alzheimer's disease (15)*.* Although we observed elevated levels of systemic IL-1β and IL-18 post-operatively, and a significant correlation was found between NLRP3 and IL-1β, IL-18, but after adjusting for confounding factors, IL-1β and IL-18 were not identified as risk factors for PND on multiple logistic regression analyses. This is inconsistent with the results of a previous study (2). The discrepancy can be ascribed to the relatively small sample size of our study, and the differences in populations and neurocognitive testing modalities between studies. Thus, further investigations in this regard are required.

Three days postoperatively, however, we found no difference in IL-1β or NLRP3 levels between PND and non-PND groups. This is because the elimination of some pathogenic factors resulted in a postoperative decline in the expressions of inflammatory cytokines. Nevertheless, the ways in which memory and cognition are affected by an inflammatory event may be modulated by time-dependent mechanisms. Probably within hours to days after inflammation, the cytokine-dependent signaling directly interacts with the mechanisms of memory events (20). The memory and cognitive deficits found in the weeks to months after anesthesia and surgery can be attributed to these primary events that initiate long-lasting neuronal changes through neurogenetic and epigenetic alterations (20).

Our results also revealed old age as a significant risk factor for PND. Older patients often have more neurovascular disease risk factors, more severe cerebral white matter injury, and less cognitive reserve than younger patients, and thus are at a higher risk of cognitive disorders after anesthesia and surgery (21). Multiple studies have identified diabetes, lower educational level, duration of surgery, and duration of ICU stay as significant risk factors for PND (21–23), but no correlation was noted in the present study. This discrepancy may be attributable to the relatively small sample size and narrow differences in the duration of ICU stay, education background, and other aspects. Hence, future research with a larger sample size is warranted. The difference in the risk factors identified among studies can also be explained by differences in the diagnosis of PND, subjective factors, and diverse analytic procedures, which may mask the effects of some risk factors.

This study has at least two limitations. First, despite the strict inclusion criteria, our study had a relatively small sample size. In addition, the relationship between the NLRP3 level and the occurrence of PND after non-cardiac surgeries is unknown. Second, our observation ended one week after the surgery because of difficulties in sampling blood and performing cognitive tests, and therefore, the temporal change in NLRP3 after surgery and the long-term relationship between NLRP3 and PND remain unknown.

## Conclusions

In this study, high serum NLRP3 level at the end of surgery was associated with a higher risk of cognitive dysfunction seven days after heart surgery. Larger clinical studies are required to provide more robust evidence of the value of NLRP3 as a predictive biomarker for PND.

## Data Availability

The original contributions presented in the study are included in the article/Supplementary Material, further inquiries can be directed to the corresponding author/s.
